# Comparative Analysis of Carbohydrate Active Enzymes in the *Flammulina velutipes* var. *lupinicola* Genome

**DOI:** 10.3390/microorganisms9010020

**Published:** 2020-12-23

**Authors:** Hye-Won Yu, Ji-Hoon Im, Won-Sik Kong, Young-Jin Park

**Affiliations:** 1 Department of Medicinal Biosciences, Research Institute for Biomedical & Health Science, College of Biomedical and Health Science, Konkuk University, 268 Chungwon-daero, Chungju-si 27478, Korea; ryu1hw@kku.ac.kr; 2 Mushroom Research Division, National Institute of Horticultural and Herbal Science, Rural Development Administration, 92, Bisan-ro, Eumseong-gun 27709, Korea; jihooni24@korea.kr (J.-H.I.); wskong@korea.kr (W.-S.K.)

**Keywords:** carbohydrate-active enzyme, *Flammulina velutipes* var. *lupinicola*, whole genome sequencing

## Abstract

The purpose of this study was to determine the genome sequence of *Flammulina velutipes* var. *lupinicola* based on next-generation sequencing (NGS) and to identify the genes encoding carbohydrate-active enzymes (CAZymes) in the genome. The optimal assembly (71 kmer) based on ABySS de novo assembly revealed a total length of 33,223,357 bp (49.53% GC content). A total of 15,337 gene structures were identified in the *F.* *velutipes* var. *lupinicola* genome using ab initio gene prediction method with Funannotate pipeline. Analysis of the orthologs revealed that 11,966 (96.6%) out of the 15,337 predicted genes belonged to the orthogroups and 170 genes were specific for *F. velutipes* var. *lupinicola*. CAZymes are divided into six classes: auxiliary activities (AAs), glycosyltransferases (GTs), carbohydrate esterases (CEs), polysaccharide lyases (PLs), glycoside hydrolases (GHs), and carbohydrate-binding modules (CBMs). A total of 551 genes encoding CAZymes were identified in the *F. velutipes* var. *lupinicola* genome by analyzing the dbCAN meta server database (HMMER, Hotpep, and DIAMOND searches), which consisted of 54–95 AAs, 145–188 GHs, 55–73 GTs, 6–19 PLs, 13–59 CEs, and 7–67 CBMs. CAZymes can be widely used to produce bio-based products (food, paper, textiles, animal feed, and biofuels). Therefore, information about the CAZyme repertoire of the *F. velutipes* var. *lupinicola* genome will help in understanding the lignocellulosic machinery and in-depth studies will provide opportunities for using this fungus for biotechnological and industrial applications.

## 1. Introduction

*Flammulina velutipes* var. *lupinicola* (Physalacriaceae) was first identified in 1999 by Redhead and Petersen [1]. However, except for the following characteristics, its molecular biology and biological properties were not well known; first, basidiospores are larger (7−14.5 × 3.7–6.5 μm) than those of the typical *F. velutipes* variety. Secondly, it seems to be limited geographically (from southern to northern California) in ecologically distinctive zone (in costal dunes) largely on a specific host (*Lupinus arboreus*) native to the region. It has been suggested that *F. velutipes* var. *lupinicola* and typical varieties (*F. velutipes* var. *velutipes*) have only partial genetic differences. [1].

Enzymes, including carbohydrate esterases (CEs), glycoside hydrolases (GHs), polysaccharide lyases (PLs), glycosyltransferases (GTs), and auxiliary activities (AAs), are collectively known as carbohydrate-active enzymes (CAZymes), and these enzymes are involved in the catabolism of carbohydrates [2]. These enzymes have attracted attention in biotechnological and industrial applications as they can be used to produce bio-based products, including food, paper, textiles, animal feed and, especially, biofuels [3,4,5]. Many fungal species that exist extensively in nature, including basidiomycetes, can efficiently degrade plant lignocellulosic biomass as they possess many types of CAZymes [3,4,5]. This ability allows the fungi to inhabit a variety of natural environments. Among the various fungi present in nature, white-rotting basidiomycetes are generally known to be able to degrade both lignin and polysaccharides from plant sources [3,4,5]. Thus, the discovery and understanding of CAZymes from fungal species, including Basidiomycetes, will enable the use of these enzymes for relevant applications [3,4,5].

Currently, genome sequence analysis of various organisms is actively under way due to the advances in genome sequencing technology such as next-generation sequencing [3]. Out of many organisms that can be sequenced, several fungal species are commonly used for genome sequencing in order to discover various biomass-degrading enzymes and to understand the wood-degrading machinery in the fungal genomes [3]. For example, the genome of *Phanerochaete chrysosporium* (white rot basidiomycete) has been reported to possess a vast array of genes associated with the lignocellulolytic machinery [6]. In addition, we also previously reported the genome sequences of *Flammulina velutipes* [7], *Flammulina elastica* [8], *Flammulina fennae* [9], and *Flammulina ononidis* [10], and identified well-developed wood-degrading machineries containing various CAZymes. The study of biomass-degrading enzymes through genome sequence analysis is an active field of research to comprehensively understand the wood-degrading machinery of various fungal species in this genomic era.

In this study, to our knowledge, we have reported, for the first time, the genome sequence and a well-developed wood-degrading machinery of *F. velutipes* var. *lupinicola*. This information will potentially facilitate its applicability for biotechnological and industrial applications as well as help in understanding the potential biotechnological and industrial applications of this fungus.

## 2. Materials and Methods

### 2.1. Fungal Strain Culture and Genomic DNA Isolation

*Flammulina velutipes* var. *lupinicola* ASI4195 was obtained from the Mushroom Research Division, National Institute of Horticultural and Herbal Science (Rural Development Administration, Republic of Korea) and was grown at 25 °C on potato dextrose agar (PDA, 4 g potato starch, 20 g dextrose, 15 g agar per liter) for 15 days. For the genomic DNA extraction, the mycelia were frozen with liquid nitrogen and ground using a mortar and pestle. Extraction buffer (0.25 M Tris-HCl, 100 mM NaCl, 50 mM ethylenediaminetetraacetic acid, 5% SDS), 2 × CTAB buffer (100 mM Tris-HCl pH 8, 20 mM EDTA pH 8, 2% CTAB, 1.4 M NaCl, and 1% polyvinyl pyrrolidone), and phenol-chloroform-isoamylalcohol (25:24:1) were added to the mycelia and mixed. After centrifugation at 13.000 rpm at 4 °C for 5 min, the supernatant was mixed with 0.7 times its volume of isopropanol. This mixture was centrifuged for 15 min at 4 °C. After washing with 70% ethanol, the dried samples were dissolved in TE buffer and then treated with RNase A (Qiagen, Seoul, Korea). Final sample was quantified and validated using the NanoDrop ND1000 (NanoDrop Technologies, Inc., Wilminton, DE, USA) and the Agilent 2100 Bioanalyzer (Agilent Technologies Korea, Ltd., Seoul, Korea).

### 2.2. Genome Sequencing and Gene Modeling

Next-generation sequencing (NGS)-based genome sequencing of the *F. velutipes* var. *lupinicola* was performed using the HiSeq 2000 platform (Illumina, Inc., San Diego, CA, USA). Library preparation was performed using Paired-End DNA Sample Prep Kit (Illumina, Inc., San Diego, CA, USA) according to the manufacturer’s instructions. Raw reads (100 bp paired-end) were processed using FastQC software (http://www.bioinformatics.babraham.ac.uk/projects/fastqc/) and Trimmomatic (version 0.32) software [11] for quality checks and adapter trimmings. The resultant short-reads were used for de novo assembly using ABySS software (size = 20 to 90 kmer) [12]. Gene prediction and annotation were carried out using the Funannotate pipelines version 1.7.2 (AUGUSTUS, Codingquary, GeneMark, gilmmerhmm, and SNAP for predictions; Pfam, Uniprot, BUSCOS, Protease, and NCBI_NR for annotations) [13], which was trained using the strain, *F. velutipes* KACC42780 with transcriptome data as hints file. The predicted gene models of *F. velutipes* var. *lupinicola* were annotated using DIAMOND [14] software with the non-redundant database from the National Center for Biotechnology Information (NCBI).

### 2.3. Ortholog Clustering

*F. velutipes* var. *lupinicola* genes were analyzed by OrthoFinder (version 2.3.3) software [15] for orthologous groups clustering with the following fungal species; *F. elastica* KACC46182 [8], *F. fennae* KACC46185 [9], *F. ononidis* KACC46186 [10], *F. velutipes* KACC42780 [7], *Aspergillus nidulans* FGSC-A4 [16], *Botrytis cinerea* B05.10 [17], *Laccaria bicolor* S238N-H82 [18], *Agaricus bisporus* var. *bisporus* H97 [19], *Coprinopsis cinerea* okayama 7#130 [20], *Lentinula edodes* [21], *Cordyceps militaris* CM01 [22], *Cryptococcus neoformans* var. *grubii* H99 [23], *P. chrysosporium* RP78 [6], *Saccharomyces cerevisiae* S288C [24], *Neurospora crassa* OR74A [25], *Schizophyllum commune* H4-8 [26], *Trichoderma reesei* QM6a [27], and *Ustilago maydis* 521 [28].

### 2.4. Prediction of CAZymes and Signal Peptides

The putative genes encoding for the CAZymes, including GH, PL, CE, GT, AA, and CBM genes in *F. velutipes* var. *lupinicola,* were identified using the dbCAN meta server (http://bcb.unl.edu/dbCAN2/) including the dbCAN CAZyme domain (by HMMER search), short conserved motifs (by Hotpep search), and CAZy databases (by DIAMOND search) [29]. The predicted genes encoding for CAZymes were further processed using the SignalP 5.0 software [30] to look for signal peptides.

### 2.5. Data Availability

Raw sequencing reads were deposited in the NCBI Sequence Read Archive (SRA) under the accession number SRR9964157 (SAMN12569251, PRJNA560135).

## 3. Results and Discussion

### 3.1. Genome Sequence Assembly, Gene Modeling, and Genome Comparison

The quality checked reads (38,416,296 reads, >Q30) were derived from the raw reads (41,592,600 reads) and were used for de novo assembly using the Abyss software [12]. The resultant optimized assembly (71 kmer) consisted of 2570 sequence contigs with a total length of 33,223,357 bp (49.53% GC content) and N50 length of 48,981 bp. A total of 15,337 gene models, with an average gene length of 1122 bp, were predicted from the assembled contigs using the Funannotate pipeline [13]. The average exon and intron lengths were 24,466 and 5367 nucleotides, respectively. The general features of the *F. velutipes* var. *lupinicola* ASI4195 genome are presented in Table 1.

Out of the 15,337 predicted genes, 82.1% (12,600) had sequence similarity (0.001 > e-value) with the genes in NCBI-NR database (Table S1). The total number of genes in *F. velutipes* var. *lupinicola* was comparable to that of its closest sequenced species, *Flammulina* species, as well as to those of other basidiomycetes with a similar genome size (Table 2).

We conducted cluster analysis with other sequenced fungal species and identified 8431 groups containing at least one *F. velutipes* var. *lupinicola* protein (Table 3 and Table S2). Analysis of these clusters suggested that 47.2% of *F. velutipes* var. *lupinicola* proteins had orthologs belonging to Dikarya, and hence were conserved in basidiomycetes and ascomycetes. Among the set of homologous genes, there were 70 species-specific orthogroups containing 170 species-specific genes in *F. velutipes* var. *lupinicola* (Table 3 and Table S2).

**Figure 1:**
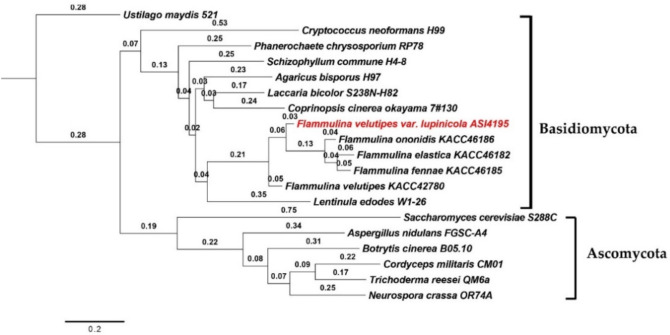
Phylogenetic analysis of fungal species based on ortholog clustering using OrthoFinder.

Ortholog analysis also revealed that, *F. velutipes* var. *lupinicola* was classified into one group with *F. ononidis*, *F. fennae*, and *F. elastica* and clustered into another group together with and *F. velutipes* and *L. edodes* by ortholog-based clustering analysis (Figure 1).

### 3.2. F. velutipes var. lupinicola and other Fungal Species CAZymes

**Figure 2:**
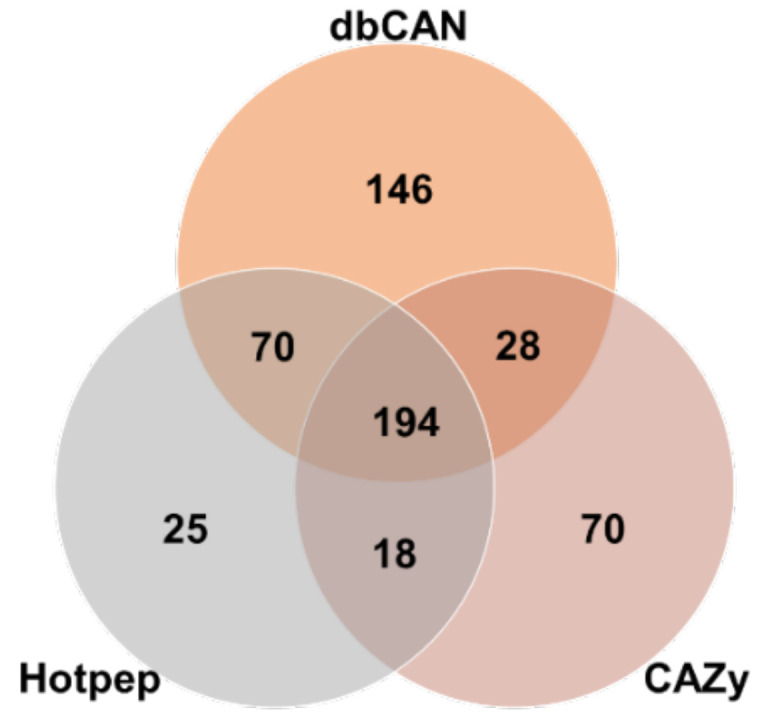
Number of identification and annotation of carbohydrate-active enzyme genes in the *Flammulina velutipes* var. *lupinicola* genome based on three different databases including the HMMER (dbCAN CAZyme domain HMM database), DIAMOND (CAZy database), and Hotpep (short conserved motifs in the PRR library database) [29].

The genome sequence of *F. velutipes* var. *lupinicola* revealed a series of genes involved in the assembly (GT) and breakdown (GHs, PLs, CEs) of carbohydrate complexes. In addition, genes related to lignin degradation (auxiliary activity; AA) and carbohydrate binding module (CBM) were identified in the *F. velutipes* var. *lupinicola* genome. Annotation of the predicted genes of *F. velutipes* var. *lupinicola* using the dbCAN meta server (http://bcb.unl.edu/dbCAN2/) [29] revealed 551 genes encoding for CAZymes, including 439 from dbCAN (HMMER), 360 from Hotpep, and 336 from the CAZy database (DIAMOND) (Figure 2 and Table 4). Among the 551 genes, some genes were annotated as different CAZymes depending on the database or predicted to simultaneously encode for two different CAZymes (Table S3). Therefore, 360–439 CAZymes, including 54–95 AAs, 145–188 GHs, 55–73 GTs, 6–19 PLs, 13–59 CEs, and 7–67 CBMs were identified in the *F. velutipes* var. *lupinicola* genome (Table S4).

#### 3.2.1. Glycosyltransferases (GTs)

GT is an enzyme that catalyzes the transfer of glycosyl groups to the specific acceptor molecules and utilizes activated donor sugar phosphates to form glycosidic bond (EC 2.4.x.y), which is involved in the biosynthesis of glycoconjugates, oligosaccharides, and polysaccharides [31,32,33,34].

**Figure 3:**
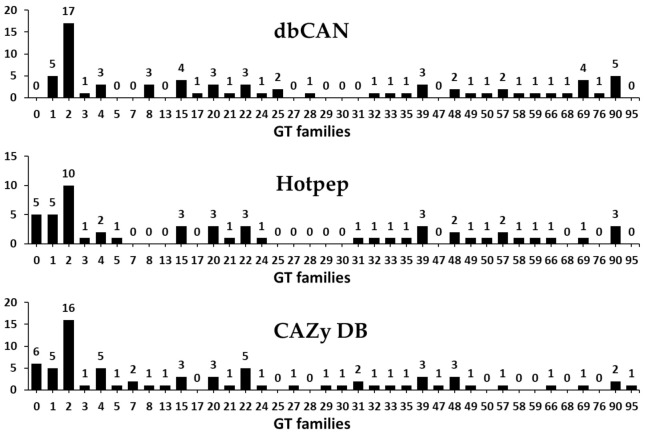
Predicted and annotated glycosyltransferase (GT) families in *Flammulina velutipes* var. *lupinicola* from dbCAN, Hotpep, and CAZy databases.

CAZyme annotation revealed that *F. velutipes* var. *lupinicola* contains a total of 55 GT families encoded by 95 genes in its genome sequence (Figure 3 and Table S3). Among the predicted GTs in the *F. velutipes* var. *lupinicola* genome, the GT2 family with 10–17 genes was the most prominent one (Figure 3 and Table S4). Several GT2 families have been identified in 18 other fungal genomes, including 12 species of basidiomycetes and six species of ascomycetes. Genome-wide comparisons of GT families indicated that the GT2 family was also prominent, suggesting that the GT2 family is a major component of the GT family in most fungal species (Table S4). Moreover, genome sequencing of bacterial, archaeal, and eukaryotic organisms has revealed that there are a large number of genes encoding GTs (about 1–2% of gene products) in their genomes [2]. Breton et al. [31] suggested that the number of families might increase with the incorporation of newly discovered GT genes, and not all sequences encoding GT were present in public databases. At the time of writing (August 2020), GT2 and GT4 were listed in the CAZy database and account for about half of the total number of GTs, with more than 740,000 classified and 17,000 unclassified GT sequences classified into 111 families (CAZy database; http://www.cazy.org/).

GT, which is a membrane protein located mainly in endoplasmic reticulum and Golgi apparatus, has a C-terminal catalytic domain, an N-terminal cytoplasmic tail, and a signal-anchor domain that consists of a non-cleavable signal peptide [35,36]. Thus, the signal peptides predicted in 6 out of the 95 GT genes in the in *F. velutipes* var. *lupinicola* genome suggest that these signatures are likely to act as signal-anchor domains (Table S3).

Previous studies have demonstrated the difficulty in classifying GTs based on sequence similarity as many GTs have different activities even though the GT sequences are very similar [37]. In this study, six genes from the *F. velutipes* var. *lupinicola* genome were annotated as GT0 family (not yet assigned to the family) based on amino acid sequence similarities (Table S3). This indicates that further studies based on structural and mutational analysis of these genes annotated with the GT0 family are needed to characterize their enzymatic properties.

#### 3.2.2. Carbohydrate Esterases (CEs)

CEs represent a class of esterases which catalyze *N*-de- or *O*-deacylation to remove esters from substituted saccharides and are widely used as biocatalysts in industrial processes as well as in biotechnological applications [38,39,40]. CEs have a wide variety of substrate specificities, such as specificity for feruloyl-polysaccharide (feruloyl esterases, EC 3.1.1.73), xylan (acetylxylan esterases, EC 3.1.1.72), acetic ester (acetyl esterases, EC 3.1.1.6), peptidoglycan (poly-*N*-acetylglucosamine deacetylases, chitin (chitin deacetylases, EC 3.5.1.41), EC 3.5.1.104), and pectin (pectinesterase, EC 3.1.1.11) [41]. These CEs are currently classified into 18 families in the CAZy database (CAZy database; http://www.cazy.org/), including more than 87,000 classified and 1700 non-classified CEs.

**Figure 4:**
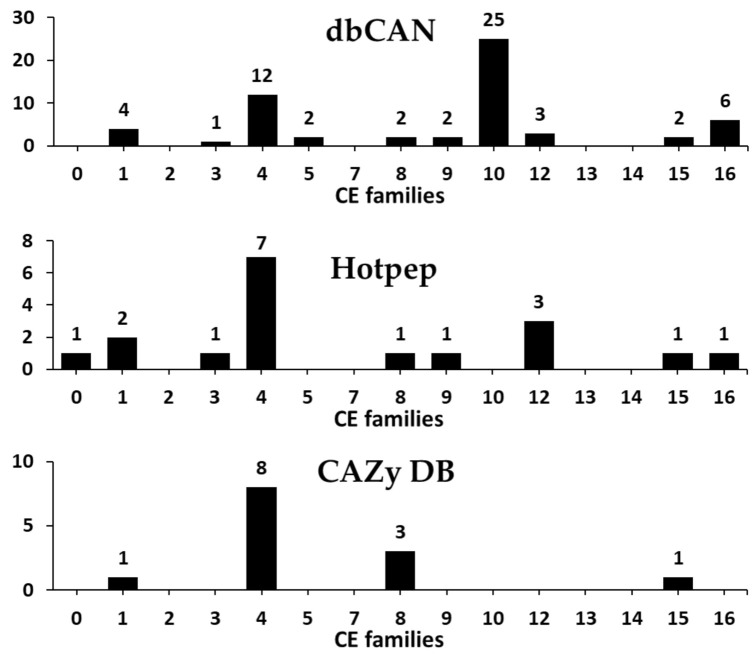
Predicted and annotated carbohydrate esterase (CE) families in *Flammulina velutipes* var. *lupinicola* from dbCAN, Hotpep, and CAZy databases.

Our results revealed a total of 61 predicted CEs classified into 11 families in the *F. velutipes* var. *lupinicola* genome based on the dbCAN meta server search (Figure 4 and Table S4). CE10 family was prominent, with 25 CEs, and the CE4 family was the second largest family with 12 CEs in the *F. velutipes* var. *lupinicola* genome (Figure 4). Genome-wide comparisons of CEs revealed that the total number of CEs in *F. velutipes* var. *lupinicola* was similar to that found in other basidiomycetes, including *Flammulina species*, *C. cinerea*, and *S. commune*, with 57 to 63 CEs (Table S4). In addition, CE1, −4, and −16 families are prominent in several basidiomycetes (Table S4). Furthermore, basidiomycetes and ascomycetes were found to vary in the number of CE families, as only five CEs are known in *Cryptococcus neoformans* and two in *S. cerevisiae* (Table S4). Although CAZyme predictions have found a number of genes encoding for the CE10 family members in the *F. velutipes* var. *lupinicola* genome, most members of the CE10 family have been reported to act on non-carbohydrate substrates [2,42].

Among the many enzymes identified and classified as CE, some characteristic features have been identified in the amino acid sequence. Members of the CE1, CE4, CE5, and CE7 families have been reported to possess the GXSXG (Gly-Xaa-Ser-Xaa-Gly) conserved motif, as well as the Ser-His-Asp catalytic triad. It has been also reported that CE2 and CE3 family members possess the Gly-Asp-Ser-(Leu) (GDS (L)) motif in their amino acid sequence. In addition, CE16 family members also possess the Ser-Gly-Asn-His catalyst residues and GDS (L) catalytic motif [43]. In the present study, 12 CEs were found to possess conserved motifs, such as GXSXG (Table 5). Some CE family members have been found to have Gly-Xaa-Xaa-Leu (GXXL) motifs, which are generally found in esterases that show high homology to class C β-lactamases [44,45]. In addition, in the present study, 11 of the 12 genes that were predicted to encode CE4 family members were found to have a conserved sequence (Phe-Asp-Asp-Gly-Pro) and further studies are needed to elucidate the biochemical role of this motif (Table 5).

#### 3.2.3. Glycoside Hydrolases (GHs)

**Figure 5:**
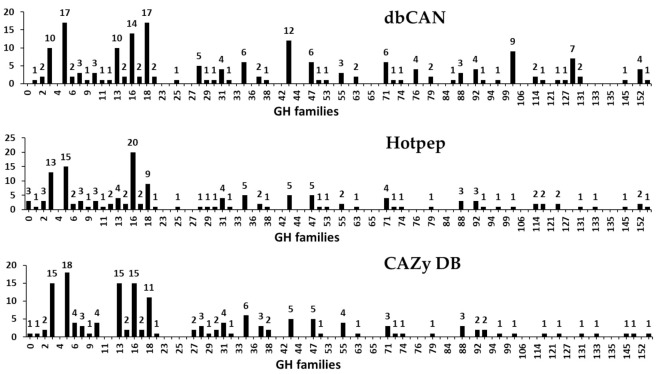
Predicted and annotated glycoside hydrolase (GH) families in *Flammulina velutipes* var. *lupinicola* from dbCAN, Hotpep, and CAZy databases.

GHs (glycosidases or glycosyl hydrolases, EC 3.2.1.x) catalyze the hydrolysis of glycosidic bonds of complex carbohydrates such as cellulose, hemicellulose, and starch [46,47]. Previously, Henrissat [47] classified GHs into 35 families by comparing 301 amino acid sequences. Currently, 168 families with more than 874,000 classified and 17,000 unclassified GH sequences are listed in the CAZy database (http://www.cazy.org/). In the present study, a total of 246 GHs classified into 55 families were predicted in the *F. velutipes* var. *lupinicola* genome based on the dbCAN meta server search (Figure 5 and Table S4). GH family classification also revealed that the GH16 family was the most prominent one with 20 genes in the *F. velutipes* var. *lupinicola* genome as those in other fungal species. In addition, multiple copies of GH5 and GH18 found in the *F. velutipes* var. *lupinicola* genome were similar to those in other basidiomycetes (Figure 5 and Table S4).

GH16 family comprises a number of enzymes with known activities, such as lichenase (EC 3.2.1.73), xyloglucan xyloglucosyltransferase (EC 2.4.1.207), agarase (EC 3.2.1.81), κ-carrageenase (EC 3.2.1.83), endo-β-1,3-glucanase (EC 3.2.1.39), endo-β-1,3-1,4-glucanase (EC 3.2.1.6), and endo-β-galactosidase (EC 3.2.1.103). Most of these enzymes have the conserved motif, Glu-Xaa-Asp-Xaa-(Xaa)-Glu (EXDX[X]E), and the two glutamic acid (E) residues have been reported to be important for their catalytic activities [48,49,50]. Likewise, all of the predicted GH16 family members in *F. velutipes* var. *lupinicola* also possessed this conserved motif (EXDX[X]E) (Table 6).

Although many GHs have been reported to have signal sequences as they are either secreted or targeted to other cellular locations such as the periplasmic space or Golgi apparatus, signal peptide prediction revealed that 80 out of 246 GHs had signal peptides in their amino acid sequence (Table S3). In previous studies, approximately one-third of GH genes have been reported to have no signal sequence, and hence, GHs without signal sequences indicate their cellular location such as the periplasmic space or Golgi apparatus [51].

Polysaccharides in plant cell walls often form complex structures and synergistic action of enzymes is required to efficiently degrade such complex structures. GHs are essential for the processing of polysaccharides (chitin, cellulose, and xylan from plant), which represent a major source of carbon and nitrogen in nature [2,52]. Substrate specificity is one of the distinctive features of these enzymes, which include cellulases (GH5, -6, -7, -8, -9, -12, -44, -45, and -48), xylanases (GH10, -11, and -30), chitinases (GH18, -19, and -85) and they can act on cellulose, xylose, and chitin, respectively. Another group of enzymes, β-glucosidases (GH1 and -3) can convert cellobiose into glucose [2,52]. In this study, CAZyme annotation revealed that *F. velutipes* var. *lupinicola* contains a series of genes associated with cellulase (GH5, -6, -7, -9, and -12), xylanase (GH10, -11, and -30), chitinase (GH18 and -85), and β-glucosidases (GH1 and -3) in its genome sequence (Figure 5 and Table S3).

Recently, sequenced bacterial genomes have revealed the variability of GHs and their potential for industrial degradation of biopolymers [53,54,55]. In addition, fungi also show high levels of hydrolytic activity involved in polysaccharide degradation in nature, and the degradation machineries of many species have been characterized to evaluate their potential in biotechnological applications [56,57,58]. Therefore, more than 200 genes encoding various GHs in the *F. velutipes* var. *lupinicola* genome suggesting that this fungus has great potential for biotechnological applications.

#### 3.2.4. Polysaccharide Lyases (PLs)

**Figure 6:**
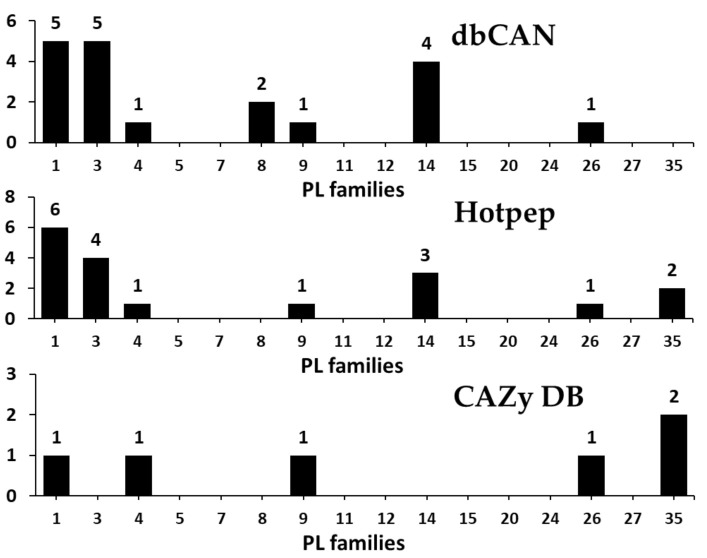
Predicted and annotated polysaccharide lyase (PL) families in *Flammulina velutipes* var. *lupinicola* from dbCAN, Hotpep, and CAZy databases.

PL (Eliminase, EC 4.2.2.-) cleaves uronic acid-containing polysaccharides through a β-elimination mechanism to produce unsaturated polysaccharides and is currently classified into 40 families in the CAZy database [2,59]. In the present study, a total of 22 PLs classified into 8 families were predicted in the *F. velutipes* var. *lupinicola* genome based on the dbCAN meta server search (Figure 6 and Table S4). Among them, the PL1 family was the most prominent, and three families, including PL4, -9, and -26, consisted of only one PL (Figure 6 and Table S4). Some of the PL family members were reported to be phylum specific [60]. Our results showed that while other Basidiomycetes had high numbers of genes encoding for PL14 family members in their genomes, PL20 was only found in ascomycetes, and PL14 appeared to be specific to Basidiomycota. Additionally, although PL5, -15, and -24 family members are Basidiomycota specific, they are present only in a few species of Basidiomycetes (Table S4).

Pectate (EC 4.2.2.2 and EC 4.2.2.9) or pectin lyases (EC 4.2.2.10) can degrade polygalacturonan (PGA) and pectate lyases are mainly produced by bacterial species [61,62]. However, fungal species produce both pectate and pectin lyases [62]. Genome sequencing of several fungal species, including basidiomycetes, has revealed a number of genes encoding PL, which has led to their potential for use in biotechnological applications. It has been reported that *S. commune* (basidiomycete) not only produces higher levels of pectinase than *Aspergillus niger* (ascomycete) in wheat bran, but also high levels of polygalacturonase [26,63].

Pectin and pectate lyases have been classified into 6 PL families, PL1, -2, -3, -9, and -10, in the CAZy database [2]. All of the characterized fungal pectate lyases belong to the families PL1, PL3, and PL9 and the pectin lyases belong to PL1 family [2]. Our results showed that the genes encoding for PL family members, including families 2 and 10, were absent in the *F. velutipes* var. *lupinicola* genome or in other fungal species (Figure 6 and Table S4). Additionally, the majority of PLs were pectate lyases (PL1 and -3) in the *F. velutipes* var. *lupinicola* genome. Interestingly, most basidiomycetes lack PL family member 9, whereas *Flammulina* species and *S. commune* were found to have only the PL9 family (Table S4). These results suggest that *F. velutipes* var. *lupinicola* might be a potential candidate for future research focused on polysaccharide lyase as the number of genes encoding PL family members 1, 3, and 9 is similar to that of *S. commune*.

#### 3.2.5. Auxiliary Activities (AAs)

Lignin degradation enzymes such as lytic polysaccharide monooxygenases (LPMOs), involved in depolymerization of non-carbohydrate components (lignin), are classified into AA families [2,64]. In addition, these members originally classified as GH61 and CBM33 have also been found to be involved in the depolymerization of lignin and are now reclassified as AA families [2,64]. AA members are classified into a total of 16 families, including ligninolytic enzymes (9 families) and lytic polysaccharide monooxygenases (6 families), and till date, more than 15,000 classifieds and 50 unclassified AAs have been identified based on amino acid sequence similarities [2].

**Figure 7:**
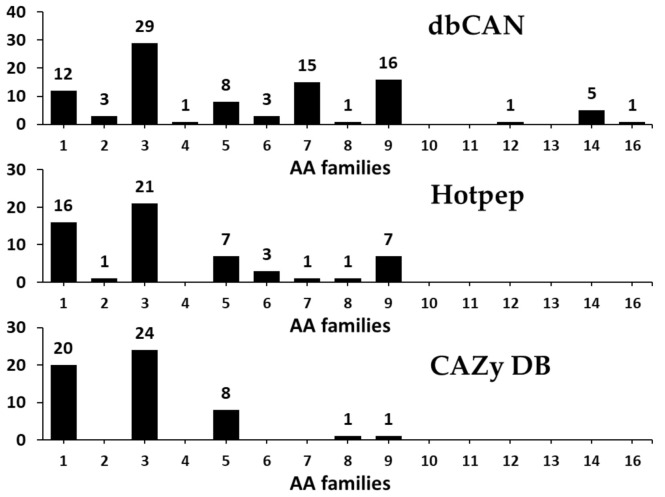
Predicted and annotated auxiliary activities (AA) families in *Flammulina velutipes* var. *lupinicola* from dbCAN, Hotpep, and CAZy databases.

In the present study, CAZyme annotation revealed that *F. velutipes* var. *lupinicola* contains a total of 12 AA families with genes for 104 AAs in its genome sequence (Figure 7 and Table S4). AA family classification also revealed that AA3 is the major representative of the AA family, with 29 AA3 family members (glucose-methanol-choline oxidoreductase, cellobiose dehydrogenase, alcohol oxidase, aryl-alcohol oxidase/glucose oxidase, pyranose oxidase), and AA1 (multicopper oxidases, laccase) comprising the second largest families, with 20 AAs in the *F. velutipes* var. *lupinicola* genome (Figure 7 and Table S4). Interestingly, the total number of AAs in the *F. velutipes* var. *lupinicola* genome was found to be similar to *C. cinerea* but different from other *Flammulina* species, and there were also more AA1 families than other basidiomycetes (Table S4).

In previous studies, some of the AAs were found to possess the conserved motifs required for interaction with the substrate, and this was particularly seen in case of laccases (EC 1.10.3.2, AA1 family), which were found to have His-Xaa-His (HXH), His-Xaa-His-Gly (HXHG), His-Xaa-Xaa-His-Xaa-His (HXXHXH), and His-Cys-His-Xaa^3^-His-Xaa^4^-Met/Leu/Phe (HCHXXXHXXXXM/L/F) motifs in their amino acid sequences [65]. Similarly, GMC oxidoreductase proteins (AA3 family) have also been reported to possess a conserved motif such as β-α-β dinucleotide binding-motif (Gly-Xaa-Gly-Xaa-Xaa-Gly-Xaa^18^-Glu) that interacts with the flavin adenine dinucleotide cofactor [66,67,68]. Our results also showed to that copper-binding and β-α-β dinucleotide-binding motifs are present in the 9 (AA1 families) and 16 genes (AA3 families) of *F. velutipes* var. *lupinicola*, respectively, indicating that these genes may act as laccases and GMC oxidoreductases (Table 7).

Degradation of wood by fungi usually begins with the depolymerization of lignin, which accelerates further degradation of wood polymers due to highly reactive lignin radicals [69,70]. Therefore, our results, including extensive identification of the AA family in the of *F. velutipes* var. *lupinicola* genome, suggest that this fungus can be potentially used for the production of biomaterials such as biofuels in the future.

#### 3.2.6. Carbohydrate-Binding Modules (CBMs)

Generally, the amino acid sequences which possesses carbohydrate binding activity in a carbohydrate-active enzyme is known as CBM, and this can bind to the carbohydrate ligands in order to enhance the catalytic activity of a carbohydrate-active enzyme such as GH, PL, and GT [71,72]. Moreover, CBM is often found in proteins without hydrolytic activity, which are known as scaffoldins and they help in organizing the catalytic subunits into non-covalent multi-protein complexes (cellulosome) [72]. Till date, CBMs have been classified into 87 families in the CAZy database, which includes more than 237,000 classifications and 800 non-classified CBM sequences [2].

**Figure 8:**
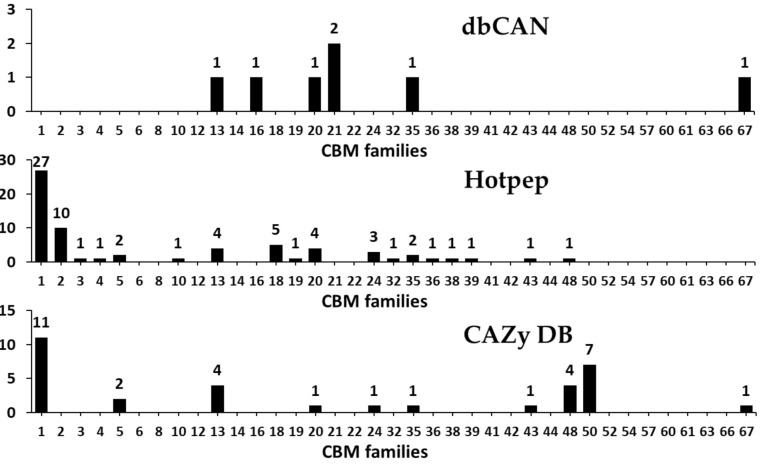
Predicted and annotated carbohydrate-binding module (CBM) families in *Flammulina velutipes* var. *lupinicola* from dbCAN, Hotpep, and CAZy databases.

In the present study, we found that a total of 22 CBM families with genes for 80 CBMs in the *F. velutipes* var. *lupinicola* genome based on the dbCAN meta server search (Figure 8 and Table S4). The distribution of CBM, along with multiple copies of CBM1, -13 and -50 family members, is similar to that found in other fungal species (Figure 8 and Table S4). In addition, we found differences in the abundance of some CBM family members between basidiomycetes and ascomycetes, and particularly, CBM 18 family members were found in ascomycetes more than in other basidiomycetes, as well as CBM 12 family members were not observed in all ascomycetes such as in *F. velutipes* var. *lupinicola* (Figure 8 and Table S4). These results are consistent with the results of a previous study by Zhao et al. [60], which reported that ascomycetes generally have fewer members of CBM 5 and -12 family than basidiomycetes, while they have more members of CBM 18 family. CBMs have been reported to be required for the activity of cellobiohydrolases classified into the GH6 and -7 families [60]. In the present study, CBM 1 family members were found in the GH6 or GH7 members identified in the *F. velutipes* var. *lupinicola* genome (Table S3). In addition, other GH families were found to possess CBMs in their genes, suggesting that these CAZymes may require CBM to efficiently degrade their substrates (Table S3).

## 4. Conclusions

In the present study, we extensively investigated the lignocellulolytic machinery in basidiomycete fungus *F. velutipes* var. *lupinicola*.

We sequenced the genome of *F. velutipes* var. *lupinicola* and identified the following genes involved in lignocellulosic biomass degradation; 54~95 auxiliary activities enzymes, 145–188 glycoside hydrolases, 55–73 glycosyltransferases, 6–19 polysaccharide lyases, 1–59 carbohydrate esterases, and 7–67 carbohydrate binding-modules. Although more detailed studies are needed, this CAZyme repertoire of *F. velutipes* var. *lupinicola* suggests that this fungus might be applied to produce various biomaterials, including bioethanol through consolidated bioprocessing (CBP), an effective processing method for production of bioethanol from lignocellulosic biomass.

## Figures and Tables

**Table 1:** *Flammulina velutipes* var. *lupinicola* genome sequencing statistics.

Hiseq 2000 NGS Analysis	Total Reads (100 bp)	41,592,600
	Reads After Trimming (%), >Q 30	38,416,296 (92.36)
De Novo Assembly	Optimized hash value (kmer)	71
	Total number of contigs (Depth of coverage)	2570 (110)
	Number of contigs (≥1 kb)	1658
	Contig N50 (bp)	48,981
	Length of longest contig (bp)	814,722
	Total bases in contigs (bp)	33,223,357
	Total bases in contigs (≥1 kb)	32,579,599
	GC content (%)	49.53
Gene Prediction	Predicted gene	15,337
	Average gene (bp) and protein (aa) length	1122 and 486.40
	Average exon per gene	4.59
	Average exon and intron size (bp)	244.66 and 53.6

**Table 2:** Comparison of the genome characteristics of *Flammulina velutipes* var. *lupinicola* and other basidiomycetes.

Fungal Species	*F. Velutipes var. lupinicola*	*F. ononidis*	*F. fennae*	*F. elastica*	*F. velutipes*	*L. bicolar*	*C. cinerea*	*P. chrysosporium*	*U. maydis*	*S. commune*	*L. edodes*
**Strain**	ASI4195	KACC 46186	KACC 46185	KACC 46182	KACC 42780	S238N-H82	Okayama 7#130	RP78	521	H4-8	W1-26
**Genome (Mb)**	33.22	34.5	32.4	35	35.6	60.71	36.19	35.15	19.6	38.67	48.3
**Genes**	15,337	12,269	11,591	12,536	12,218	23,132	13,393	13,602	6785	16,319	14,002

**Table 3:** Ortholog analysis of *Flammulina velutipes* var. *lupinicola* and other fungal species.

Fungal Species		Total Number of Genes	Number of Genes in Orthogroups Containing Species (%)	Number of Genes in Species-Specific Orthogroups (%)	Number of Genes Unassigned to Any Orthogroups (%)	Number of Orthogroups Containing Species (%)	Number of Species-Specific Orthogroups
**Basidiomycota**	*F. velutipes var. lupinicola* ASI4195	15,337	14,563 (96.5)	170 (1.1)	526 (3.5)	8431 (47.2)	70
	*Flammulina fennae* KACC46185	11,591	11,318 (97.6)	33 (0.3)	273 (2.4)	7631 (42.7)	15
	*Flammulina ononidis* KACC46186	12,269	11,948 (97.4)	67 (0.5)	321(2.6)	7896 (44.2)	29
	*Flammulina elastica* KACC46182	12,536	12,079 (96)	104 (0.8)	457 (4)	7856 (44.0)	41
	*Flammulina velutipes* KACC42780	12,218	10,957 (90)	199 (1.6)	1261 (10)	7088 (39.7)	81
	*Agaricus bisporus var. bisporus* H97	10,438	9496 (91)	1284 (12.3)	942 (9)	5960 (33.4)	184
	*Coprinopsis cinerea okayama* 7#130	13,393	11,646 (87)	1945 (14.5)	1747 (13)	6821 (38.2)	421
	*Cryptococcus neoformans var. grubii* H99	6967	5931 (85)	322 (4.6)	1036 (15)	4740 (26.5)	83
	*Laccaria bicolor* S238N-H82	23,132	20,555 (89)	7400 (32)	2577 (11)	8202 (45.9)	1478
	*Lentinula edodes* W1-26	14,002	11,862 (85)	947 (6.8)	2140 (15)	6688 (37.4)	290
	*Phanerochaete chrysosporium* RP78	13,602	11,236 (83)	1480 (10.9)	2366 (17)	6650 (37.2)	358
	*Schizophyllum commune* H4-8	16,319	13,423 (82)	2642 (16.2)	2896 (18)	7104 (39.8)	597
	*Ustilago maydis* 521	6785	5674 (84)	227 (3.3)	1111 (16)	4765 (26.7)	74
**Ascomycota**	*Aspergillus nidulans* FGSC-A4	10,680	1349 (87)	268 (2.5)	1349 (13)	6383 (35.7)	111
	*Botrytis cinerea* B05.10	16,447	6677 (59)	587 (3.6)	6677 (41)	6533 (36.6)	210
	*Cordyceps militaris* CM01	9651	1162 (88)	191 (2)	1.162 (12)	6323 (35.4)	53
	*Neurospora crassa* OR74A	10,785	1809 (83)	501 (4.6)	1809 (17)	6453 (36.1)	183
	*Saccharomyces cerevisiae* S288C	6002	1790 (73)	443 (6.7)	1790 (27)	3525 (19.7)	136
	*Trichoderma reesei* QM6a	9115	1404 (86)	167 (1.7)	1404 (14)	6580 (36.8)	51

**Table 4:** CAZymes of *Flammulina velutipes* var. *lupinicola* and other fungal species.

Taxon	Species	CAZymes						No. of CAZyme (Annotation DB)	Reference
		AA	GH	GT	CE	CBM	PL		
**Basidiomycota**	***F. velutipes* var. *lupinicola***	95	188	71	59	7	19	439 (Hmmer dbCAN)	This study
		57	145	55	18	67	18	360 (Hotpep)	
		54	157	73	13	33	6	336 (CAZy database)	
	*F. fennae*	86	220	85	57	45	20	513	[9]
	*F. ononidis*	87	228	87	61	40	21	524	[10]
	*F. elastica*	82	218	89	59	42	18	508	[8]
	*F. velutipes*	85	239	84	63	44	25	540	[8]
	*A. bisporus*	81	174	54	33	44	9	395	JGI database
	*C. cinerea*	111	195	83	60	105	16	570	[8]
	*L. bicolor*	55	170	96	18	31	7	377	JGI database
	*L. edodes*	82	254	85	44	61	11	537	[8]
	*P. chrysosporium*	85	175	65	16	62	4	407	JGI database
	*S. commune*	78	241	85	57	37	18	516	[8]
	*U. maydis*	28	113	61	29	10	2	243	
	*C. neoformans*	14	97	70	5	12	4	202	CAZy database
**Ascomycota**	*C. militaris*	54	165	91	34	39	5	388	[8]
	*T. reesei*	59	210	90	32	44	5	440	[8]
	*S. cerevisiae*	5	57	68	2	12	0	144	CAZy database
	*A. nudulans*	33	267	91	30	46	23	490	CAZy database
	*N. crassa*	35	177	80	21	42	4	359	CAZy database
	*B. cinerea*	77	287	119	37	89	10	619	CAZy database

**Table 5:** Conserved motifs of CE families in *Flammulina velutipes* var. *lupinicola*.

CE Family	Gene	Motifs
CE1, 4, 5		GXSXG, SHD, FDDGP *
CE1	g5266	GDSLG
	g5267	GDSLG
	g8160	GDSLG, SHD
CE4	g374	GDSDG, FDDGP
	g2000	FDDGP
	g4109	FDDGP
	g7304	FDDGP
	g7333	GTSEG, FDDGP
	g8346	FDDGP
	g10068	GPSFG, FDDGP
	g10591	GDSNG
	g13055	FDDGP
	g13320	GSSSG, FDDGP
	g14347	GDSAG, FDDGP
	g14354	GDSAG, FDDGP
CE5	g4988	GWSQG
	g15044	GWSQG
CE3, 16		GDS(L)
CE3	g4116	GDS
CE16	g5620	GDS
	g5623	GDS
	g5624	GDS
	g8182	GDS
	g15048	GDS

* Asterisk indicates CE family 4 specific motif.

**Table 6:** Conserved motifs of GH16 family in *Flammulina velutipes* var. *lupinicola*.

GH Family	Gene	Motifs
		EXDX[X]E
**GH16**	g2766	EIDLE, EIDLIE
	g9518, g12441	EIDVE
	g11166	EIDWE
	g14715	EADDE
	g311, g4184, g7248, g7679, g8220, g9061, g10939	EIDIIE
	g3620, g9596	EIDVFE
	g4175	EVDIGE
	g4746, g5694, g7094, g12591, g14715	EIDIFE
	g4760	EIDIME
	g5774, g11509, g12838, g14181	EIDILE
	g7259	EIDVLE
	g11952	EVDILE
	g11992	EIDIVE

**Table 7:** Conserved motifs of GH families in *Flammulina velutipes* var. *lupinicola.*

AA Family	Gene	Motifs			
**AA1**		**HXHG**	**HXH**	**HXXHXH**	**HCHXXXHXXXXM/L/F**
	g658	HWHG	HSH	HPFHLH	HCHIDWHLEAGL
	g2787	HHHG	HGH	HPFHFH	HCHIEWHLEVGL
	g4337	HWHG	HSH	HPFHLH	HCHIDWHIEAGL
	g4797	HWHG	HSH	HPFHLH	HCHVDWHMEAGL
	g8250	HWHG	HSH	HPFHLH	HCHIDWHLEIGL
	g8252	HWHG	HSH	HPFHLH	HCHIDWHLDIGL
	g10087	HWHG	HSH	HPFHLH	HCHIDWHLELGL
	g12086	HGHG	HAH	HPIHKH	HCHVSQHAAGGM
	g12686	HHHG	HAH	HPFHLH	HCHIEWHLEVGL
**AA3**		**GXGXXGX^18^E**			
	g1272	GGGTAGLALAARLSE-DSNTTVLVLE			
	g2268	GAGLAGTTVAARLAE-DAGVSVLLIE			
	g2484	GSGSAGSIIATRLAE-DPNVSVCLLE			
	g7746	GGGTAGLVVAARLSE-DPNTSVLVLE			
	g8286	GGGTAGLILGARLSE-DSDTTVLVLE			
	g8482	GAGPGGSTVANRLTE-DPSLSVLLVE			
	g9880	GGGTAGVTLATRLAE-DGTHTVGVIE			
	g10382	GGGIGGAVVANRLTE-TSSVNVLLLE			
	g11534	GAGTAGSVVANRLTE-DRNVTVLVLE			
	g11997	GAGSAGMVVATRLAE-NPDASVCIIE			
	g12094	GAGTAGLVLARRLSE-KTSLKVGVIE			
	g13184	GGGAAGAVIANRLTE-IDTFSVLILE			
	g13185	GGGAAGAVVANRLTE-IDRFSVLVLE			
	g13756	GAGTAGNVVAARLSE-NRNMSVLVIE			
	g13888	GGGTAGLIIAARLSE-NADTTVLVLE			
	g14144	GGGTAGLIIAARLSE-NADTTVLVLE			

## Supplementary Materials

The following are available online at https://www.mdpi.com/2076-2607/9/1/20/s1, Table S1: Gene prediction and annotation of *Flammulina velutipes* var. *lupinicola*, Table S2: Ortholog analysis of *Flammulina velutipes* var. *lupinicola* with other sequenced fungal species, Table S3: *Flammulina velutipes* var. *lupinicola* CAZymes identified from three different databases, Table S4: Distribution of CAZymes in the *Flammulina velutipes* var. *lupinicola* genome and in other fungal genomes.
